# Cysteamine exerts in vitro antiviral activity against the SARS-CoV-2 Delta and Omicron variants

**DOI:** 10.1038/s41420-022-01080-8

**Published:** 2022-06-15

**Authors:** Tonino Alonzi, Alessandra Aiello, Federica Repele, Laura Falasca, Massimo Francalancia, Anna Rosa Garbuglia, Giovanni Delogu, Emanuele Nicastri, Mauro Piacentini, Delia Goletti

**Affiliations:** 1 National Institute for Infectious Diseases “L. Spallanzani”-IRCCS, Rome, Italy; 2 Institute of Microbiology, Università Cattolica del Sacro Cuore - Fondazione Policlinico Gemelli, Rome, Italy; 3 Mater Olbia Hospital, Olbia, Italy; 4 Department of Biology, University of Rome “Tor Vergata”, Rome, Italy

**Keywords:** Viral infection, Mechanisms of disease

## Abstract

The novel SARS-CoV-2 variants of concern (VOC) represent a considerable global alarm because their mutations are known to affect transmissibility and cause immune escape. While preventing severe disease and deaths, the available vaccines do not avoid infection; therefore, COVID-19 disease management still requires effective therapies. We have recently reported that the aminothiol cysteamine, a drug already applied to humans, exerts direct antiviral activity against SARS-CoV-2 and has in vitro immunomodulatory effect. To evaluate whether this compound exerts antiviral effects also against SARS-CoV-2 variants, we performed different infected cell-based assays using Wild type, Delta, or Omicron VOC. We found that cysteamine significantly reduces the cytopathic effect induced by SARS-CoV-2 Wild type strain and Delta variant in Vero E6 cells. On the other hand, cysteamine had no effects on the survival of cells infected with the Omicron variant, due to the lack of cytotoxicity on Vero E6 cells, at least when infected at MOI = 0.001 for 72 h. Moreover, cysteamine significantly reduced the production of Wild type, Delta, and Omicron variants as measured by the virus released in the culture media (Vero E6 and Calu-3 cells) and by transmission electron microscopy analysis (Vero E6 cells). Notably, cysteamine is more effective in inhibiting the Omicron rather than Delta or Wild type viruses, with an 80% inhibition of Omicron production compared to 40% of Wild type and Delta variant. Overall, our findings demonstrate that cysteamine exerts direct antiviral actions against SARS-CoV-2 Delta and Omicron variants, in addition to the Wild type virus. Our data further demonstrate that cysteamine is a good candidate as repurposing drug for the treatment of SARS-CoV-2 infection for the present and, likely, the future VOC and, therefore, it would be important to investigate its clinical relevance in randomized clinical trials.

## Introduction

In the last two years, the world lifestyle and economic activities have been dreadfully influenced by the COVID-19 pandemic outbreak [1]. Indeed, SARS-CoV-2 infection caused over 6.0 million deaths worldwide in addition to half-billion affected people with various degree of clinical outcome. Although an incredible effort has been made to control the COVID-19 pandemic by vaccination, which limited the clinical severity and death outcome, the mutations of the virus to variants with high transmission is increasing the time required for the eradication of the infection [1, 2]. SARS-CoV-2 shows a great capacity of changing genetics and at least 13 variants have been reported so far, with mutations localized in the amino acids present at the N-terminus and the receptor-binding region. The most spread variants have been Alpha, Beta (B.1.351), Gamma (P.1), Delta (B.1.617.2), and Omicron (B.1.1.529), with Delta and Omicron being the most alarming ones [3, 4]. Recently, a new variant with the Delta backbone and Omicron spike has been described [5]. Among all the SARS-CoV-2 variants, Omicron has generated more worries due to its fast transmission rate. In fact, Omicron is much more contagious than Wild type SARS-CoV-2 or as infectious as Delta [6]. The newly emerged Omicron BA.2 is believed to be even more contagious, although the higher contagiousness remains so far unexplained. One of the major concerns about the Omicron variants is related to the evidence that the available vaccines do not prevent infection, although they prevent severe disease and deaths [7].

COVID-19 still needs more effective therapies [8]. For instance, human monoclonal antibodies (mAbs) do not work in advanced severe disease and some of them were found to be ineffective against the emerging variants of the virus, in particular for the Omicron variant [9]. Repurposing drugs can be a valid pragmatic approach to treat diseases as COVID-19 [10–14]. We have recently shown that in vitro cysteamine, an approved drug for nephropathic cystinosis, (i) reduces SARS-CoV-2-induced cytopathic effects (CPE) in Vero E6, (ii) decreases the viral production in Vero E6 and Calu-3 cells and (iii) displays anti-inflammatory effects in cells from COVID-19 patients [15]. Cysteamine, 2-aminoethanethiol, is a rather simple aliphatic compound, which became, since the FDA’s approval in 1994, the gold standard of care for the treatment of nephropathic cystinosis [16, 17]. Cystinosis is a genetic autosomal recessive disease of lysosomes characterized by progressive renal failure in children [16].

In this study, we evaluated the impact of cysteamine on reducing the SARS-CoV-2 cytopathic effects and replication among the different SARS-CoV-2 variants of concern (VOC). Our results demonstrate a very promising, potent effect of cysteamine in preventing viral production and cytotoxicity of the Omicron variant.

## Results and Discussion

### Cysteamine significantly reduces the cytopathic effect induced by SARS-CoV-2 Wild type and Delta variant in Vero E6 cells

Recently, we have reported that cysteamine and its oxidation product cystamine exert a direct antiviral action against the Wild type SARS-CoV-2 strain isolated at INMI L. Spallanzani IRCCS [15]. We then tested whether cysteamine had an impact on the life cycle of recent SARS-CoV-2 VOC and, therefore, we performed the viral-induced cytopathic effects (CPE) inhibition assay in Vero E6 cells.

Cells were treated for 1 h with either cysteamine, using 2-fold serial dilutions ranging from 1000 μM to 125 μM, or with the vehicle (H_2_O at 1, 0.5, 0.25, or 0.125% v/v) as control, before SARS-CoV-2 infection with Wild type, Delta or Omicron variants (MOI = 0.001). Cells were cultured in the presence of the drug, which was added to the medium every 24 h after infection until the end of the experiments, 72 h post-infection (p.i.).

**Fig. 1:**
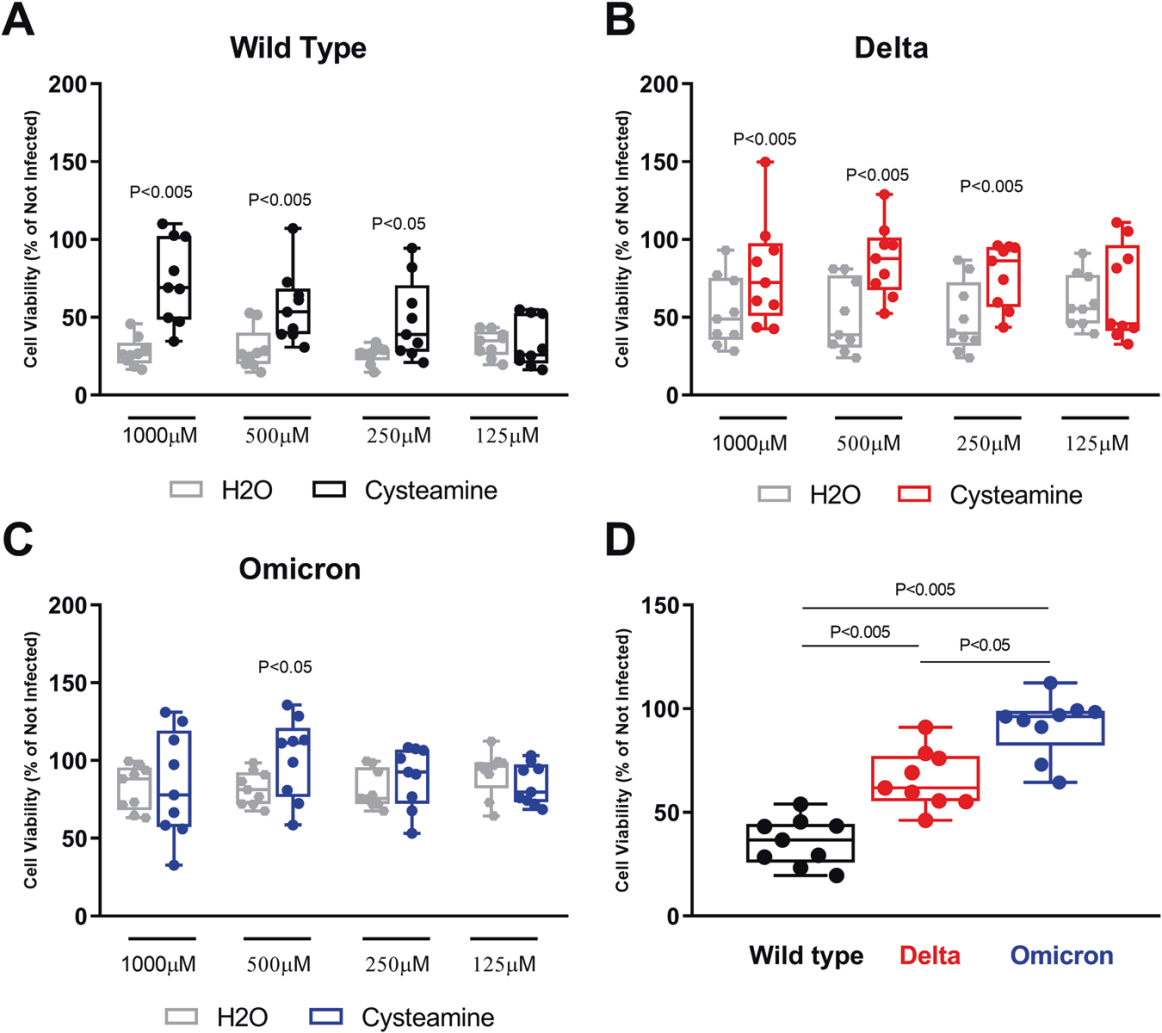
Cysteamine inhibits CPE induced by SARS-CoV-2 variants in Vero E6 cells. Vero E6 cells were treated with different doses of cysteamine or H_2_O as indicated and infected with SARS-CoV-2 wild type strain (**A**), Delta (**B**), and Omicron (**C**) variants (MOI = 0.001). The percentage of surviving cells was evaluated by crystal violet staining assay 72 h post infection (h.p.i.). The results were reported setting not infected cells as 100% and calculating as a relative value the percentage of not infected cells in the different experimental conditions. (**D**) The different infectious ability of the SARS-CoV-2 variants in Vero E6 cells was expressed comparing the percentage of not infected cells based on the CPE detection. Statistical analysis was performed using Wilcoxon matched-pairs rank test and Friedman test. Data are expressed as median ± S.D. (*n* = 9) of three independent experiments performed in triplicate.

As shown in Fig. 1, cysteamine significantly prevented the SARS-CoV-2-induced CPE in a dose-dependent manner in cells infected with either the Wild type virus (Fig. 1A) or Delta variant (Fig. 1B). On the other hand, cysteamine had no effects on the survival of cells infected with the Omicron variant (Fig. 1C). This discrepancy between Omicron and the other viruses is due to the lack of cytotoxicity exerted by the Omicron variant on Vero E6 cells, when infected at a MOI = 0.001 for 72 h (Fig. 1D; blue box). Interestingly, the Delta variant induced an intermediate degree of cell death between the Wild type and Omicron variant viruses (Fig. 1D; red box).

### Cysteamine significantly reduces the production of Delta and Omicron variants in Vero E6 and Calu-3 cells

To understand whether cysteamine directly affects the life cycle of the different SARS-CoV-2 variants, we measured the amount of the infectious viral particles released in the culture medium of Vero E6 cells treated with either cysteamine or with the drug-vehicle (H_2_O) at 72 h.p.i. We evaluated viral production by back-titrating the culture supernatants of the infected cells using a limiting dilution assay, as we recently described [15].

**Fig. 2:**
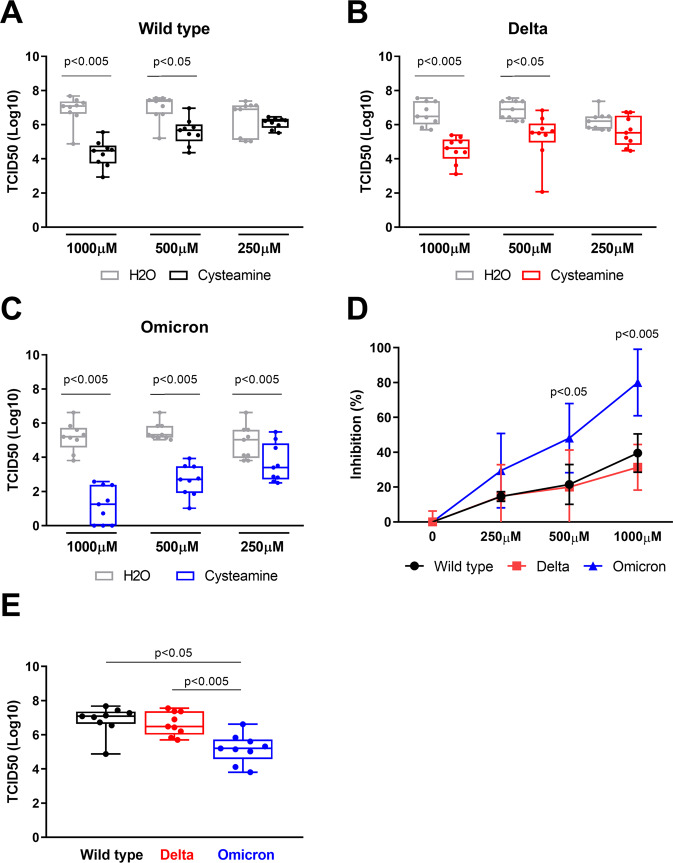
Cysteamine decreases the production of SARS-CoV-2 variants in Vero E6 cells. Supernatants from Vero E6 cells SARS-CoV-2 infected with wild type strain (**A**), Delta (**B**) and Omicron (**C**) variants, treated with different doses of cysteamine or H_2_O as indicated were harvested 72 h.p.i.. Virus yield was measured by back-titration based on detection of CPE and compared among the wild type strain, Delta and Omicron variants in absence of cysteamine (**E**). Infectious titers were calculated with the Reed–Muench method and expressed as 50% tissue-culture effective dose (TCID50) values. (**D**) The efficacy of the cysteamine treatment in Vero E6 cells was expressed as percentage of inhibition of the virus yields measured in panels **A**–**C**. Statistical analysis was performed using Wilcoxon matched-pairs rank test. Data are expressed as median ± S.D. (*n* = 9) of three independent experiments performed in triplicate.

Notably, cysteamine significantly reduced the yield of infectious SARS-CoV-2 Wild type and Delta when used at 1000 and 500 μM (Fig. 2A, B). On the other hand, cysteamine significantly reduced the viral production in cells infected with the Omicron variant at all the concentrations tested (Fig. 2C), thus suggesting that it is more effective in inhibiting Omicron rather than Delta or the Wild type viruses. In fact, as shown in Fig. 2D, cysteamine decreased up to 80% of the Omicron viral production in Vero E6 cells, compared to 40% of Wild type and Delta variant.

Interestingly, although Vero E6 cells were infected by the different VOC at the same MOI, Omicron-infected cells generated a significantly lower viral yield compared to that generated by cells infected with either Wild type or Delta viruses (Fig. 2 panel E) as expected [18], thus confirming that Omicron has a decreased replicative fitness, at least in Vero E6 cells.

Omicron viral production is lower than that found using Wild type or Delta and, as reported above, Omicron does not induce a CPE in Vero E6 cells. Therefore, it is tempting to speculate that the decreased viral production is responsible for the lower viral cytotoxicity. However, the Delta variant, although displaying a similar viral yield (Fig. 2 panel E), induced lower CPE values compared to those of the Wild type virus (Fig. 1 panel D). These results indicate that Delta and, likely, Omicron variants are less cytopathic for the cells when compared with the Wild type virus.

We also evaluated by transmission electron microscopy analysis the impact of cysteamine (1000 µM) at 48 h.p.i. of Vero E6 cells. The time was chosen to perform the experiment before the virus-mediated CPE was evident.

**Fig. 3:**
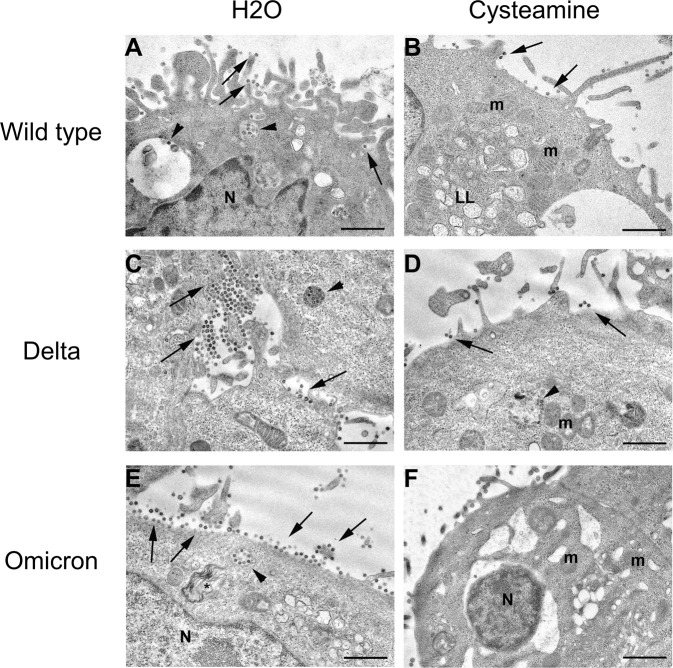
Electron microscopy images of Vero E6 cells infected with SARS-CoV-2 variants. Representative electron micrographs of Vero E6 cells at 48 h.p.i. with different SARS-CoV-2 variants, in the absence (**A**, **C**, **E**) or in the presence (**B**, **D**, **F**) of cysteamine treatment. Cells infected with Wild type (**A**), Delta (**C**), and Omicron (**E**) display similar findings: numerous viral particles are visible attached to the cell surface (arrows) and inside membrane-bound vesicles in the cytoplasm (arrowheads). Vacuoles are present in the cytoplasm, many of them consisting in lipolysosomes (LL), and convoluted membranes vesicles (*). Cysteamine treatment completely blocks the infection with Omicron (**F**), while part of Wild type (**B**) and Delta (**D**) treated cells show the presence of viral particles, although reduced in number compared with the untreated cells. N nucleus, m mitochondria. Scale bars: 1 µm.

In the untreated cells, the three SARS-CoV-2 viruses did not show significant differences in their infecting capability (Figs. 3A, C, E). A great number of viral particles were observed along the plasma membrane of cells infected with Wild type (Fig. 3A), Delta (Fig. 3C), or Omicron (Fig. 3E) variants. Viral particles (presenting black dots, due to cross-section through the viral nucleocapsid) were found as clusters attached to microvilli of the cell surface, even in the intercellular space between the neighboring cells of the monolayer (Fig. 3C). For all the three strains considered, numerous intracellular particles were found enclosed within membrane-bound vacuoles; in addition, the majority of infected cells displayed cytoplasmic vacuoles, lipolysosomes and convoluted membranes vesicles (Fig. 3A, C, E) [19].

On the other hand, cysteamine treatment induced a reduction of the number of viral particles with a different efficacy among the SARS-CoV-2 variants. As previously reported [15], in Wild type-infected cells only 30% of cysteamine-treated cells displayed viral particles when compared to untreated cultures. Cysteamine-treated cells presented a reduced number of viruses at the cell surface and a lower number of intracytoplasmic vacuoles containing virions (Fig. 3B). Cysteamine had a lower effect in cells infected with Delta variant with about 50% of the cells still showing viral presence; importantly, although the number of the particles attached to the cell surface appeared reduced, cytoplasmic vacuoles containing viruses were found (Fig. 3D). Notably, by ultrastructural observations we found that cysteamine reduced Omicron variant infection in the majority of the cells. A limited number of cells displayed large vacuoles; few mature viral particles were visible either along the cell surface or inside the cytoplasmic compartments (Fig. 3F).

**Fig. 4:**
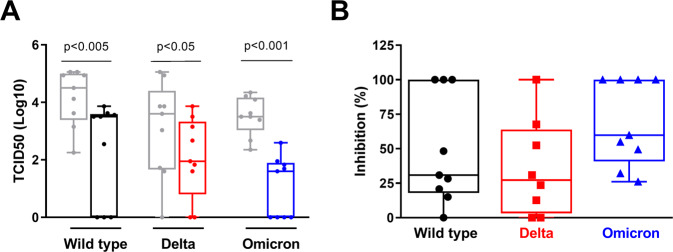
Cysteamine decreases the production of SARS-CoV-2 variants in Calu-3 cells. (**A**, **B**) Calu-3 cells were treated with cysteamine (1000 µM) or H_2_O (1% v/v) 1 h before SARS-CoV-2 infection (MOI = 0.01) as indicated. (**A**) Virus yield of culture media from SARS-CoV-2-infected cells was measured by back-titration as described in the methods. Infectious titers were calculated with the Reed–Muench method and expressed as 50% tissue-culture effective dose (TCID50) values. (**B**) The efficacy of the cysteamine treatment in Calu-3 cells was expressed as percentage of inhibition of the virus yields measured in panel A. Statistical analysis was performed using Wilcoxon matched-pairs rank test. Data are expressed as median ± S.D. (*n* = 9) of three independent experiments performed in triplicate.

We next asked whether cysteamine exerts the antiviral activity against the SARS-CoV-2 VOC also in the lung-derived epithelial cellular model Calu-3. Cells were treated for 1 h with either cysteamine (1000 µM) or H_2_O (1% v/v) as control, before SARS-CoV-2 infection (MOI = 0.01) and then cultured in the presence of the drug, which was added to the medium 24 h after infection until the end of the experiments (48 h.p.i.), when the culture media were collected and viral titers measured. As shown in Fig. 4, cysteamine significantly reduced viral production in Calu-3 cell cultures, thus suggesting that this compound exerts a potent direct antiviral effect also in these human cells, which are similar to the natural targets of SARS-CoV-2.

Overall, the decreased viral-induced CPE (i.e. Wild type and Delta variant), the reduced viral production, and the lower infected cell number evaluated by the electron microscopy analysis demonstrated that cysteamine significantly decreased the replication of SARS-CoV-2 of both Delta and Omicron VOC, in addition to the Wild type virus, as previously reported [15].

The ongoing COVID-19 pandemic rapidly spread around the world, resulting in enormous consequences in health, economic, and social aspects. Although the vaccination has allowed a return to near-normal life, there is still an urgent need to develop new therapies to counter the pandemic, especially in light of the high rate of viral mutations with, consequently, the increased risk of immune viral escape.

Herein, we presented evidence that cysteamine, an already approved safe drug for cystinosis, elicits effective anti-SARS-CoV-2 activity in vitro. We showed that cysteamine retained its potency against the two major relevant variants Delta and Omicron, suggesting a broad activity against the different SARS-CoV-2 isolates.

Since cysteamine can interact with both viral and host proteins, it may act influencing many extracellular and/or intracellular processes; therefore, it is difficult to draw definitive conclusions on the mechanism(s) responsible for the observed results.

It has been reported that disulfide-thiol balance is important for the viral entry of different enveloped viruses such as SARS-CoV, HIV, and murine hepatitis virus, thus suggesting that cysteamine may decrease SARS-CoV-2 infectivity, at least in culture [20–22]. This hypothesis is corroborated by some evidence showing how thiol-based chemical probes decrease both SARS-CoV-2 production in human nasal epithelial (HNE) cells and ACE2 receptor binding via allosteric disulfide disruption of the spike glycoprotein [23, 24]. Moreover, preliminary results reported by Khanna and colleagues showed that thiol drugs such as Mesna, bucillamine, cysteamine, and WR-1065, inhibit cell entry of several SARS-CoV-2- VOC [19].

On the other hand, viruses depend on and hijack host cellular machinery to replicate. It is plausible to hypothesize that important factors for viral life cycle that influence the cellular microenvironment may be affected by the action of cysteamine. For instance, the redox cellular state plays a key role in the regulation of many essential signalling pathways including cell death and proliferation, and modulates the expression of several redox-sensitive genes, which can be exploited by viruses [25]. In this regard, SARS-CoV-2 decreases in Vero E6 cells the levels of cellular thiols, lowering the reduced form of glutathione (GSH), a potent intracellular antioxidant, and increasing the extracellular levels of cysteine, thus demonstrating that also SARS-CoV-2 favours its replication by promoting a pro-oxidant environment in the host cells [26]. Notably, cysteamine stimulates the transport of cysteine into cells by promoting GSH synthesis [27, 28], thus supporting the hypothesis that the free thiol group of cysteamine can react with thiols or disulfide bound of proteins, modulating/altering their function.

Autophagy is another fundamental cellular mechanism influenced by cysteamine, known to be involved in the SARS-CoV-2 replication and pathogenesis [17, 29]. In particular, coronaviruses exploit the autophagic machinery to form double-membrane vesicles necessary for viral replication and inhibit the process to escape clearance by the host cells [30, 31]. Overall, it is reasonable to hypothesize that cysteamine, targeting many cellular enzymes thanks to its ability to modify the disulfide bonds or susceptible cysteine residues, influences proteins involved in one or more intracellular processes required for the replication and pathogenesis of SARS-CoV-2.

Furthermore, we provided indication that cysteamine also acts as an immunomodulator of SARS-CoV-2 specific response, at least in vitro [15]. These data are further strengthened by the preliminary results by Khanna and colleagues reporting that cysteamine reduces the inflammation of the lungs of SARS-CoV-2-infected Syrian hamster [19]. If confirmed, these results are the support of the rationale to propose clinical trials of thiol drugs for COVID-19 therapy.

## Materials and methods

### Cell lines, cysteamine, and viruses

Kidney epithelial Vero E6 cells, derived from an African green monkey (Chlorocebus sp.; formerly called *Cercopithecus aethiops*), were cultured in Minimum Essential Medium (MEM) (Merck Life Science, Milan, Italy; Cat. No. M2279) supplemented with 2 mM L-glutamine, 1% penicillin/streptomycin solution (Euroclone, Milan, Italy; Cat. No. ECB3000D; ECB3001D, respectively) and 10% fetal bovine serum (FBS) (Gibco, Life Technologies Italia, Monza, Italy; Cat. No. 10270106), previously inactivated at 56 °C for 30 min. Calu-3 cells, derived from human epithelial lung adenocarcinoma, were grown in RPMI 1640 (Euroclone, Milan, Italy; cat. n°ECB9006L) supplemented with heat-inactivated 10% FBS, 2 mM L-glutamine, and 1% penicillin/streptomycin solution. All cell lines were maintained at 37 °C with a 5% CO_2_ humidified atmosphere.

Cysteamine (CAS 60-23-1; Merck Life Science, Milan, Italy; Cat. No. M9768) was purchased as powder, and was subsequently solubilized in deionized water as 100 mM concentrated solution, filtered using a 0.22 µm membrane, and finally used at the indicated concentrations by dilution in medium. To ensure the activity of the drug, the solution was made fresh each time prior to use.

The different SARS-CoV-2 viruses used in all the experiments performed with Vero E6 and Calu-3 cells were isolated at the National Institute for Infectious Disease (INMI) L. Spallanzani IRCCS: Wild type strain (2019-nCoV/Italy-INMI1; GenBank MT066156) [32]; Delta strain [hCoV-19/Italy/LAZ-INMI-648isl/2021(GISAID Accession ID EPI_ISL_3230211)]; Omicron strain [hCoV-19/Italy/LAZ-INMI-2890/2021 (GISAID Accession ID EPI_ISL_7716384)].

### Virus protection assay

The antiviral activity of cysteamine was tested by the SARS-CoV-2-induced cytopathic effect (CPE) inhibition assay performed in a biosafety level 3 facility using Vero E6 cells. The day before infection, Vero E6 cells were washed with 1x phosphate buffered saline (PBS) (Euroclone, Milan, Italy; Cat. No. ECB4004L), detached with trypsin-EDTA solution (Merck Life Science, Milan, Italy; Cat. No. T3924), and 2.5 × 10^4^ cells/well were seeded into 96-well flat-bottom plates in a 100 µL final volume of MEM medium. Plates were then incubated overnight to allow cell adherence. The day after, cell culture medium was discarded and cells were treated with two-fold serial dilutions of cysteamine (ranged 1000 µM−25 µM) or of the drug-vehicle [H_2_O ranged 1–0.125% (v/v)] and incubated for 1 h (h) at 37 °C in a 5% CO_2_ atmosphere. Vero E6 cells were then infected with the Wild type strain or Delta or Omicron variants at 0.001 multiplicity of infection (MOI; which reflects the ratio of PFU to the number of infected cells), using MEM supplemented with heat-inactivated 2% FBS and 2 mM L-glutamine in presence of cysteamine or H_2_O, and incubated for 1 h at 37 °C and 5% CO_2_. Supernatants were then carefully discarded and replaced by fresh medium containing the same concentrations of either cysteamine or H_2_O. In the following 72 h, cells were treated by adding the compound/control to the culture medium every 24 h and were maintained at 37 °C with 5% CO_2_. After 72 h, microplates were observed by light microscope for the presence of CPE. Then, supernatant was discarded and 100 µL of crystal violet solution (Merck Life Science, Milan, Italy; Cat. No. 9448-2.5L-F) containing 2% formaldehyde (Carlo Erba reagents, Milan, Italy; Cat. No. 415666) were added to each well for 20 min. Subsequently, the fixing solution was removed, plates washed with tap water, and then immersed in a bath of 2% formaldehyde solution in 1x PBS for further 20 min. Finally, cell viability was evaluated with a photometer measuring the optical density (OD) at 595 nm and reported as the percentage of surviving cells compared to the uninfected cells.

### SARS-CoV-2 production yield assay

The ability of cysteamine to reduce the replication of the SARS-CoV-2 variants was evaluated through a virus yield production assay. To this aim, SARS-CoV-2 viral particles released in culture medium after infection of both Vero E6 and Calu-3 cells were back-titrated by limiting dilution assay on Vero E6 cells.

To evaluate the viral production in Vero E6 cells, the supernatants harvested from the cells infected for the CPE assay were back-titrated by serial dilutions in three replicates using MEM supplemented with heat-inactivated 2% FBS and 2 mM L-glutamine and added in 96-well plates containing 2.5 × 10^4^ Vero E6 cells/well.

To evaluate the viral production in Calu-3 cell line, 2.5 × 10^5^ cells per well were seeded into 24-well plates in a final volume of 1 mL of RPMI medium, and then left overnight at 37 °C to allow adherence. At 24 h post-seeding, media were changed with those containing cysteamine (1000 µM) or H_2_O [1% (v/v)] as control, and cells incubated for 1 h. Then, Calu-3 cells were infected with either the Wild type strain or Delta and Omicron variants at MOI = 0.01 using RPMI supplemented with heat-inactivated 2% FBS and 2 mM L-glutamine in presence of either cysteamine or H_2_O, and incubated at 37 °C and 5% CO_2_. One-hour post infection, supernatants were removed, cells washed with 1x PBS and cultured with fresh medium containing the treatments as above. Cells were then treated at 24 h with the drugs and incubated at 37 °C with 5% CO_2_ until 48 h.p.i., when supernatants were collected and back-titrated as reported for Vero E6 cells.

Virus back-titration was evaluated based on CPE detection, and infectious titers were reported as 50% tissue-culture effective dose (TCID50) values, calculated according to the Reed–Muench method.

### Transmission Electron Microscopy

SARS-CoV-2 infected Vero E6 and Calu-3 cells were observed by transmission electron microscopy (TEM) using standard procedures. To this aim, Vero E6 cells were seeded into 8-well chamber slides at density of 7.5 × 10^4^ cells per well and processed as described above. For TEM analysis, cells were treated only with cysteamine at 1000 µM or H_2_O [1% (v/v)] as control. After 48 h, supernatant was discarded, cell washed with 1x PBS, and then fixed for 4 h with 2.5% glutaraldehyde in 0.1 M cacodylate buffer at 4 °C. Cells were then treated with 0.25% glutaraldehyde in 0.1 M cacodylate buffer and left at 4 °C until processed. Subsequently, samples were dehydrated in graded ethanol and embedded in Epon resin, as previously described [33, 34]. Ultrathin sections were stained with 2% uranyl acetate and observed with a transmission electron microscope (JEOL JEM 2100 Plus, Japan Electron Optics Laboratory Co. Ltd., Tokyo, Japan). Images were digitally captured with a digital camera TVIPS (Tietz Video and Image Processing Systems GmbH, Gauting, Germany).

### Statistical analysis

GraphPad Prism 7.04 software (GraphPad, San Diego, CA, USA) was used to analyse data. The following non-parametric inference tests were applied: the Friedman test was used for comparisons among groups and the Wilcoxon matched-pairs rank test for pairwise comparisons with Bonferroni correction when appropriate.

## Data Availability

The data supporting the finding of this study are available from the corresponding author upon request.

## References

[1] Chen J, Lu H, Melino G, Boccia S, Piacentini M, Ricciardi W (2020). COVID-19 infection: the China and Italy perspectives. Cell. Death Dis.

[2] Forni G, Mantovani A, COVID-19 Commission of Accademia Nazionale dei Lincei, Rome. (2021). COVID-19 vaccines: where we stand and challenges ahead. Cell Death Differ.

[3] DeGrace MM, Ghedin E, Frieman MB, Krammer F, Grifoni A, Alisoltani A (2022). Defining the risk of SARS-CoV-2 variants on immune protection. Nature.

[4] Karim SSA, Karim QA (2021). Omicron SARS-CoV-2 variant: a new chapter in the COVID-19 pandemic. Lancet.

[5] Kreier F (2022). Deltacron: the story of the variant that wasn’t. Nature.

[6] Menni C, Klaser K, May A, Polidori L, Capdevila J, Louca P (2021). Vaccine side-effects and SARS-CoV-2 infection after vaccination in users of the COVID Symptom Study app in the UK: a prospective observational study. Lancet Infect Dis.

[7] Madhi SA, Kwatra G, Myers JE, Jassat W, Dhar N, Mukendi CK (2022). Population immunity and Covid-19 severity with Omicron variant in South Africa. N. Engl J Med.

[8] Ferraccioli G, Gremese E, Goletti D, Petrone L, Cantini F, Ugel S (2022). Immune-guided therapy of COVID-19. Cancer. Immunol Res.

[9] Vitiello A, Ferrara F, Auti AM, Di Domenico M, Boccellino M. Advances in the Omicron variant development. J Intern Med. 2022. 10.1111/joim.13478. 10.1111/joim.13478 PMC9115048 35289434

[10] Goletti D, Cantini F (2021). Baricitinib therapy in Covid-19 Pneumonia - An unmet need fulfilled. N. Engl J Med.

[11] Cantini F, Niccoli L, Nannini C, Matarrese D, Natale MED, Lotti P (2020). Beneficial impact of Baricitinib in COVID-19 moderate pneumonia; multicentre study. J Infect.

[12] Cantini F, Niccoli L, Matarrese D, Nicastri E, Stobbione P, Goletti D (2020). Baricitinib therapy in COVID-19: A pilot study on safety and clinical impact. J Infect.

[13] Pecetta S, Pizza M, Sala C, Andreano E, Pileri P, Troisi M (2021). Antibodies, epicenter of SARS-CoV-2 immunology. Cell Death Differ.

[14] Petrone L, Petruccioli E, Alonzi T, Vanini V, Cuzzi G, Najafi Fard S (2021). In-vitro evaluation of the immunomodulatory effects of Baricitinib: Implication for COVID-19 therapy. J Infect.

[15] Alonzi T, Aiello A, Petrone L, Najafi Fard S, D’Eletto M, Falasca L (2021). Cysteamine with in vitro antiviral activity and immunomodulatory effects has the potential to be a repurposing drug candidate for COVID-19 therapy. Cells.

[16] Topaloglu R (2021). Nephropathic cystinosis: an update on genetic conditioning. Pediatr Nephrol.

[17] Villella VR, Esposito S, Ferrari E, Monzani R, Tosco A, Rossin F (2019). Autophagy suppresses the pathogenic immune response to dietary antigens in cystic fibrosis. Cell Death Dis.

[18] Zhao H, Lu L, Peng Z, Chen LL, Meng X, Zhang C (2022). SARS-CoV-2 Omicron variant shows less efficient replication and fusion activity when compared with Delta variant in TMPRSS2-expressed cells. Emerg Microbes Infect.

[19] Khanna K, Raymond W, Jin J, Charbit AR, Gitlin I, Tang M, et al. Thiol drugs decrease SARS-CoV-2 lung injury in vivo and disrupt SARS-CoV-2 spike complex binding to ACE2 in vitro. bioRxiv. 2021;2020: 10.1101/2020.12.08.415505.

[20] Fenouillet E, Barbouche R, Jones IM (2007). Cell entry by enveloped viruses: redox considerations for HIV and SARS-coronavirus. Antioxid Redox Signal.

[21] Lavillette D, Barbouche R, Yao Y, Boson B, Cosset FL, Jones IM (2006). Significant redox insensitivity of the functions of the SARS-CoV spike glycoprotein: comparison with HIV envelope. J Biol Chem.

[22] Gallagher TM (1996). Murine coronavirus membrane fusion is blocked by modification of thiols buried within the spike protein. J Virol.

[23] Mancek-Keber M, Hafner-Bratkovic I, Lainscek D, Bencina M, Govednik T, Orehek S (2021). Disruption of disulfides within RBD of SARS-CoV-2 spike protein prevents fusion and represents a target for viral entry inhibition by registered drugs. FASEB J.

[24] Shi Y, Zeida A, Edwards CE, Mallory ML, Sastre S, Machado MR, et al. Thiol-based chemical probes exhibit antiviral activity against SARS-CoV-2 via allosteric disulfide disruption in the spike glycoprotein. Proc Natl Acad Sci. USA 2022;119: 10.1073/pnas.2120419119. 10.1073/pnas.2120419119 PMC8833197 35074895

[25] Liu PJ, Balfe P, McKeating JA, Schilling M. Oxygen sensing and viral replication: implications for tropism and pathogenesis. Viruses. 2020;12: 10.3390/v12111213. 10.3390/v12111213 PMC7693908 33113858

[26] Bartolini D, Stabile AM, Bastianelli S, Giustarini D, Pierucci S, Busti C (2021). SARS-CoV2 infection impairs the metabolism and redox function of cellular glutathione. Redox Biol.

[27] Wilmer MJ, Kluijtmans LA, van der Velden TJ, Willems PH, Scheffer PG, Masereeuw R (2011). Cysteamine restores glutathione redox status in cultured cystinotic proximal tubular epithelial cells. Biochim Biophys Acta.

[28] Issels RD, Nagele A, Eckert KG, Wilmanns W (1988). Promotion of cystine uptake and its utilization for glutathione biosynthesis induced by cysteamine and N-acetylcysteine. Biochem Pharmacol.

[29] Faraj J, Bodas M, Pehote G, Swanson D, Sharma A, Vij N (2019). Novel cystamine-core dendrimer-formulation rescues DeltaF508-CFTR and inhibits Pseudomonas aeruginosa infection by augmenting autophagy. Expert Opin Drug Deliv.

[30] Liang H, Luo D, Liao H, Li S (2022). Coronavirus usurps the autophagy-lysosome pathway and induces membranes rearrangement for infection and pathogenesis. Front Microbiol.

[31] Yuen CK, Wong WM, Mak LF, Wang X, Chu H, Yuen KY (2021). Suppression of SARS-CoV-2 infection in ex-vivo human lung tissues by targeting class III phosphoinositide 3-kinase. J Med Virol.

[32] Colavita F, Lapa D, Carletti F, Lalle E, Bordi L, Marsella P (2020). SARS-CoV-2 isolation from ocular secretions of a patient with COVID-19 in Italy With prolonged viral RNA detection. Ann Intern Med.

[33] Falasca L, Nardacci R, Colombo D, Lalle E, Di Caro A, Nicastri E (2020). Postmortem findings in Italian patients with COVID-19: a descriptive full autopsy study of cases with and without comorbidities. J Infect Dis.

[34] Nardacci R, Colavita F, Castilletti C, Lapa D, Matusali G, Meschi S (2021). Evidences for lipid involvement in SARS-CoV-2 cytopathogenesis. Cell. Death Dis.

